# Surface areas and adsorption energies of biochars estimated from nitrogen and water vapour adsorption isotherms

**DOI:** 10.1038/s41598-024-81030-9

**Published:** 2024-12-05

**Authors:** Kamil Skic, Agnieszka Adamczuk, Angelika Gryta, Patrycja Boguta, Tibor Tóth, Grzegorz Jozefaciuk

**Affiliations:** 1 Institute of Agrophysics, Polish Academy of Sciences, Doświadczalna 4 str, Lublin, 20-290 Poland; 2 Research Institute for Soil Sciences, Centre for Agricultural Research, HUN-REN, Fehérvári út 132-144, Budapest, Hungary

**Keywords:** Surface area, Biochar, Water vapour adsorption isotherms, Nitrogen adsorption isotherms, Adsorption energy, Cation exchange capacity, Structural materials, Bioinspired materials

## Abstract

Nitrogen adsorption isotherms, along with the BET model for interpretation, are recommended for estimating biochar surface area. The frequently measured small surface areas of biochars contrast with their high sorption and cation exchange capacities. We hypothesised that water adsorption provides a better tool for estimating the surface area of biochars. Although adsorption energy also appears to be a valuable surface characteristic, there is a lack of studies on this subject. We studied the surface areas and adsorption energies of three waste deposits – peat, willow dust and biochar prepared from these materials at different temperatures – using nitrogen and water vapour adsorption isotherms. The BET model accurately described all water vapour adsorption isotherms but failed for some nitrogen isotherms. Alternative methods for estimating surface areas and adsorption energies were proposed in cases where the BET model did not apply. Nitrogen adsorption was typically much lower than water vapour adsorption, and the estimated surface areas reflected this. However, nitrogen adsorption energies were significantly higher. Nitrogen surface areas increased with pyrolysis temperature, while water vapour surface areas decreased. The surface area estimated from nitrogen adsorption was generally much lower than needed to accommodate the surface-charged groups responsible for the cation exchange capacity of biochars.

## Introduction

In recent years, biochar, a stable carbon-rich solid bio-sourced material, has aroused broad interest due to its favourable physical and chemical properties, low-cost feedstock and excellent application potential for solving global environmental, agricultural, biomedical and energy challenges^1^. Biochar is extensively used for the remediation and improvement of soil fertility^2^, as a carrier of beneficial microorganisms^3^, and as an adsorbent of heavy metals, microplastics, or organic pollutants from wastewater^4^. The performance of biochar in different applications is mainly determined by its properties. The surface area and porosity of biochar are among the most important characteristics, which are crucial for the quantity and quality of the available active sites in biochar, thus enhancing its properties such as cation exchange capacity, water holding capacity and adsorption capacity^5^. Despite the adsorption energy of biochars appearing to be a valuable surface characteristic, according to the authors’ knowledge, there is a lack of papers on this subject. The Brunauer–Emmett–Teller method^6^ continues to be the most widely used procedure for evaluating the surface area of porous and finely-divided materials from adsorption isotherms^7^, as expressed in IUPAC recommendations^8^ and specifically addressed for biochars by the European Biochar Certificate and the International Biochar Initiative. The BET model has limitations when applied to heterogeneous materials like biochar, as it assumes that nitrogen physisorption occurs uniformly across both internal and external surfaces, an assumption that may not hold true for biochar’s complex structure^9^. One of the issues with the BET method is the selection of the linear region from the linearised BET plot^10^. Usually, the BET model is restricted to a limited region of the adsorption isotherm, typically in the *p/p*_*0*_ range of 0.05 to 0.35, under the assumption that monolayer formation will occur within this pressure range. According to Maziarka et al.^11^, the upper limit of this range is often narrowed to 0.3 or even 0.1 *p/p*_*0*_ in the case of highly complex carbonaceous materials, being valid only in the absence of microporosity and strong adsorption centres. Moreover, selecting the proper range of relative pressures should be preceded by evaluating the constant C, which describes the adsorption energy in the monolayer. A negative parameter value indicates an inappropriate selection of the *p/p*_*0*_ range, leading to an incorrect result and interpretation^12,13^. These errors affect specific surface area calculations, and most researchers do not realise the difference when using software to calculate surface area for porous materials.

Despite many limitations and uncertainties in isotherm measurements, low-temperature nitrogen adsorption is the most frequently applied method^14,15^. The use of nitrogen as an adsorbate can be problematic. The complex structure of biochars, combined with the slow diffusion of nitrogen at low measurement temperatures into small micropores and narrow constrictions similar in width to the molecular diameter of N_2_, leads to prolonged measurement times and difficulties in achieving equilibrium^16^. As a result, open hysteresis in N_2_ isotherms of biochar is often encountered, where the equilibrium between the tested material and the adsorbate is disturbed^17^. Some authors suggest that the open hysteresis at low relative pressures observed for carbon materials may result from the interaction of active surfaces with N_2_ molecules, causing the expansion or contraction of pores due to increased pressure during micropore filling^11^. In contrast, water vapour adsorption utilises a polar sorbate under analytical conditions much closer to those found in nature. Similar to nitrogen adsorption, water vapour is also encountered in specific surface area measurements of porous organic materials, including biochars^18,19^, where free hydrogen ions and carboxylic and phenolic groups localised on the surface serve as adsorption sites for water vapour molecules^14^. Wang et al.^20^ stated that very small micropores in carbon materials can adsorb water vapour, while nitrogen molecules find these adsorption centres inaccessible. Water molecules are more easily adsorbed than N_2_ on biochar surfaces and can hydrate cations in the interlayer space. However, controlling the relative water vapour pressure is much more difficult, especially at high *p/p*_*0*_ values, which is a disadvantage of water vapour adsorption compared to N_2_ adsorption and one reason N_2_ is used more often than water vapour in gas physisorption.

This paper focuses on estimating the surface area and adsorption energy of biochar from nitrogen and water vapour adsorption. The properties of biochars produced at different temperatures were compared to those of their raw substrates. Some theoretical and practical uncertainties were identified, and an attempt was made to explain them.

## Materials and methods

### Substrates

The following raw substrates were used:

- By-products from mechanical and aerobic treatment of wastewater generated during the preparation of fruit (F) and dairy (D) products and washing of individual production lines,
- Waste from an agricultural biogas plant (B) coming from the dry fermentation of corn silage (the main component), rye, apple pomace and dairy whey,
- Ordinary acidic peat (P) purchased from a garden shop, washed with 0.1 mol L^− 1^ HCl and then with distilled water by centrifuging to a pH of around 4,
- Willow sawdust.

A more detailed description of the F, D and B sludges is provided by Skic et al.^21^ and of the peat (P) in Skic et al.^22^.

The raw substrates were lyophilised and heated under different temperatures at anaerobic conditions (nitrogen flux) to produce biochars. The process was conducted in a PRC 168 × 380/80 retort furnace. Before pyrolysis, the samples placed in the furnace retort were degassed twice. After each degassing, the furnace retort was filled with working gas (nitrogen, purity 5.0) to atmospheric pressure. The samples were then heated according to the pyrolysis program. The first temperature segment involved heating the substrates to 300, 450 and 600 °C, respectively, at a rate of 600 °C·h^− 1^ with a flow rate of 5 L·min^− 1^. The second isothermal segment involved maintaining the material at the maximum set temperature for 60 min under the same type of working gas, rate of change and gas flow. The obtained biochars were used for investigations. The biochars produced from the peat were washed with HCl, as was done for the raw substrate. All studied materials are presented in Table 1.

**Table 1:** Experimental materials. The table shows materials abbreviations, which are also used in the manuscript.

	Treatment temperature °C			
Substrate	NH*	300	450	600
Fruit sludge	F	F300	F450	F600
Dairy sludge	D	D300	D450	D600
Biogas plant sludge	B	B300	B450	B600
Acidic peat	P	P300		P600
Willow wood	W	W300		W600

The empty cell means that the respective material was not prepared. *NH denotes raw, unheated substrate.

### Methods

Nitrogen adsorption isotherms were measured at 77 K using a Micromeritics 3-Flex (Norcross, GA, USA) apparatus, while water vapour was measured gravimetrically at 293.15 K in a vacuum chamber. A sample weighing approximately 1 g was used for each adsorption measurement. Both preparation procedures involved degassing under low pressure. The N_2_ method required high-temperature conditions, with samples heated overnight at 378.15 K. The gravimetric procedure involved drying the material over concentrated sulfuric acid at 293.15 K. Sulfuric acid was also used to control water vapour pressure during measurement in the drying chamber, where, during the adsorption step, the relative water vapour pressures changed due to stepwise decreasing concentrations of sulfuric acid solutions. The samples were allowed to equilibrate at the given *p/p*_*0*_ for two days. Adsorbed water vapour was computed at each stage as the difference between the weight of the sample with water and the weight of the dry sample (dried in an oven after the entire process at 378 ± 1.0 K). In the N_2_ method, the adsorption isotherm was obtained by dosing nitrogen into the sample chamber step by step. At each step, gas adsorption by the sample caused a decrease in pressure in the confined volume until equilibrium was reached. The amount of gas adsorbed was the difference between the amount introduced and the quantity filling the void volume. All measurements were performed with at least two replications.

Experimental adsorption data from the range of *p/p*_*0*_ 0.1–0.35 were fitted to the linear form of the BET equation:

1 $$x/a\left( {1 - x} \right)\,=\,x\left( {C - 1} \right)/{a_m}C{\text{ }}+{\text{ }}1/{a_m}C$$

where *x* = *p*/*p*_0_, *p* is the equilibrium pressure, *p*_0_ is the saturation pressure of the adsorbate at the temperature of adsorption, *a* [kg·kg^− 1^] is the adsorbed gas quantity at a given *x*, *a*_*m*_ [kg·kg^− 1^] is the monolayer capacity, and C is a constant related to the adsorption energy:

2 $$C\,=\,\exp (({E_a}--{E_c})/{\text{RT}}))$$

where *E*_*a*_ [J·mol^− 1^] is the energy of adsorption, *E*_*c*_ [J·mol^− 1^] is the energy of the adsorbate condensation, R [J·mol^− 1^·K^− 1^] is the universal gas constant and T [K] is the temperature.

The surface area, *S* [m^2^·g^-1^, was calculated from:

3 $$S\,=\,{a_m}{\text{L}}\omega /m$$

where L is Avogadro’s number, ω is the area occupied by a single adsorbate molecule (1.08 × 10^− 19^ m^2^ for water and 1.62 × 10^− 19^ m^2^ for nitrogen) and *m* is the adsorbent mass.

## Results and discussion

### Adsorption isotherms

**Fig. 1:**
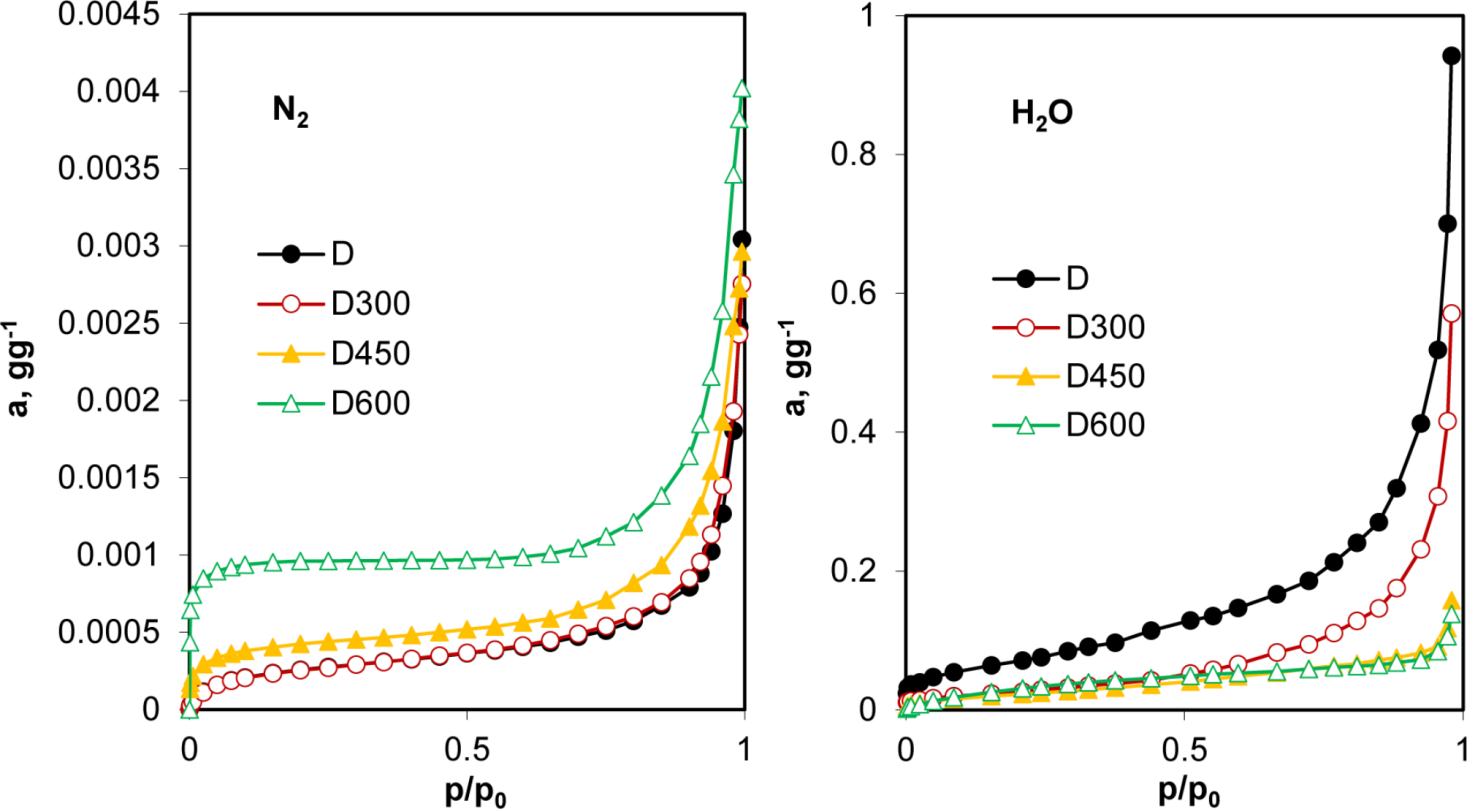
Adsorption isotherms of nitrogen at 77 K (left) and water vapour at 293.15 K (right) on dairy sludge (D) and biochars produced from it at temperatures.

Exemplary adsorption isotherms for nitrogen at 77 K and water vapour at 293.15 K on dairy sludge and biochars produced from it at various temperatures are presented in Fig. 1.

In the entire experimental window, the adsorption of nitrogen is usually much smaller (by 2 orders of magnitude) than the water vapour adsorption that has also been frequently reported in the literature^14,23^. All water vapour isotherms for the studied materials obey the BET model. In contrast, many nitrogen adsorption isotherms are not well described by this model, yielding negative values for the C constant in Eq. 1. This is true for D450 and D600 (Fig. 1), F450, F600, B600, P600 and W600, for which the isotherms are extremely steep at very low *p/p*_*0*_ values and frequently exhibit a virtually horizontal plateau that may indicate the filling of micropores of molecular dimensions^8^. The presence of a large number of such micropores in a sample may account for the significant difference between nitrogen and water molecule adsorption. Large nitrogen molecule adsorption at the micropore entrance may prevent other molecules from accessing the pore space. Such an effect may be much less pronounced for smaller water molecules. Additionally, the sample pores of various sizes may be blocked by inorganic components (ashes) produced during pyrolysis, which further affects the adsorption of large nitrogen molecules. The occurrence of this phenomenon may explain the substantial increase in the biochar surface area after so-called acid activation^24^, which leads to the dissolution and removal of the inorganic plugs. This effect may also be related to the high adsorption of nitrogen on biochar P600, which has a very high surface area (see the next paragraph) and was produced from acid-washed peat, where inorganic compounds are likely to be absent, and was again acid-washed after pyrolysis.

### Surface areas

Surface areas of the studied materials, estimated from nitrogen and water vapour adsorption isotherms using the BET model, are presented in Table 2. The application of the BET model (Eq. 1) to the D450, D600, F450, F600, B600, P600 and W600 isotherms also reveals linear ranges from which surface area estimates can be calculated.

**Table 2:** BET surface areas of the studied materials estimated from nitrogen and water vapour adsorption isotherms.

	F	F300	F450	F600	D	D300	D450	D600	B	B300	B450	B600	*P*	P300	P600	W	W300	W600
N_2_	0.99	0.96	2.27	3.44	0.74	0.73	1.09	2.24	3.5	4.22	13.4	26.4	1.06	1.4	123	0.68	1.05	1.44
H_2_O	260	124	133	120	230	90	75	105	192	91	93	93	28	174	196	163	96	87

All values are expressed in m^2^·g^− 1^. The standard deviation did not exceed 5%.

According to the BET model, the monolayer adsorption, *a*_*mon*_, is equal to the cumulative adsorption at a given *p/p*_*0*_ scaled by a factor of (1-*p/p*_*0*_). However, for the steepest nitrogen adsorption isotherms, the monolayer adsorption, even at relatively low *p/p*_*o*_ values (e.g., above 0.05), exceeds the monolayer capacity. Formally, monolayer adsorption should not exceed monolayer capacity. However, this phenomenon occurs not only for nitrogen but also for some water vapour adsorption isotherms. This likely results from an inaccurate fit of the experimental data to the BET model, uncertainties in linear BET range selection or regression errors.

To address this challenge, the surface areas were alternatively estimated from maximum monolayer adsorption, *a*_*mon, max*_, calculated as:

4 $${a_{mon,max}}=\hbox{max} \left\{ {a(p/{p_0}) \times (1 - p/{p_0})} \right\}$$

**Fig. 2:**
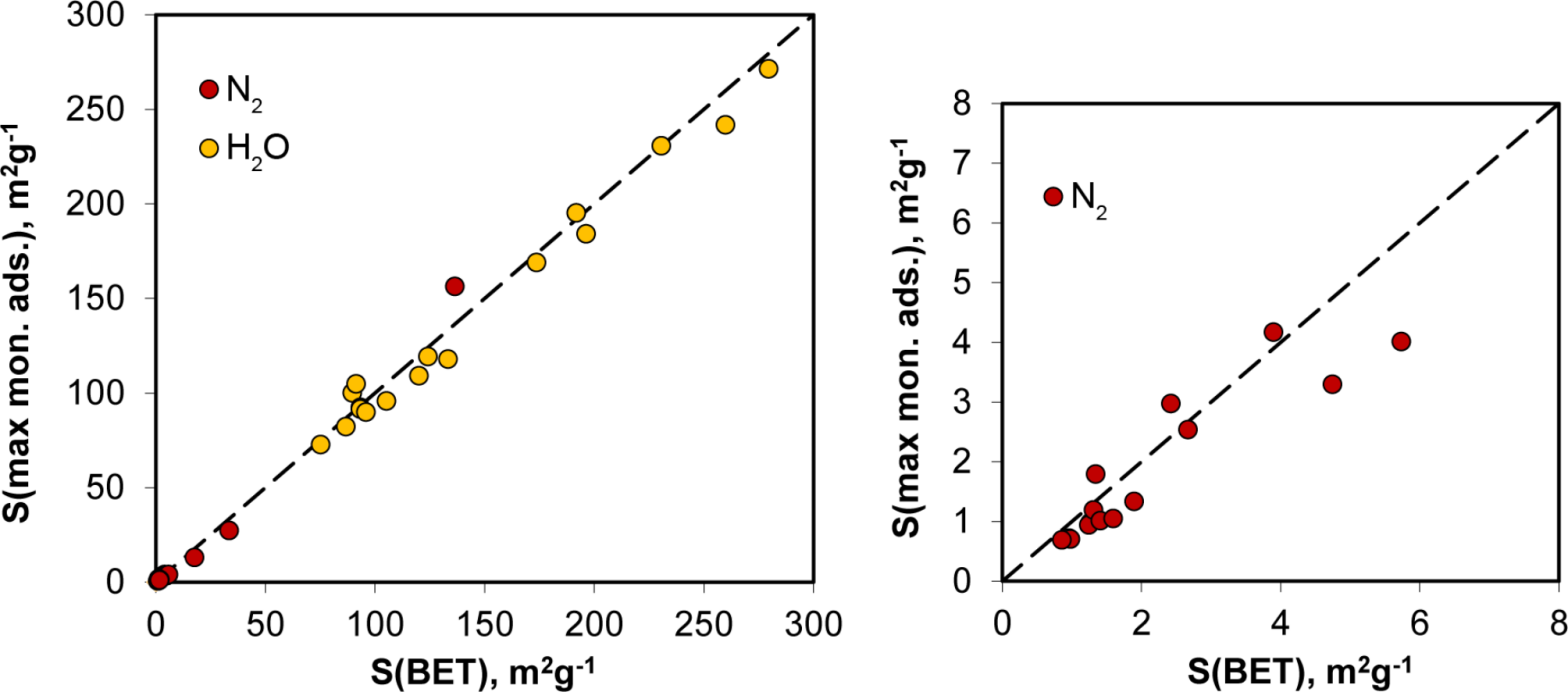
Relationship between surface areas calculated from maximum monolayer adsorption (Eq. 4) and the linear BET equation (Eq. 1) for the studied samples. The dashed line represents a 1:1 relationship. The chart on the right displays nitrogen surface areas, which are not distinguishable due to their much smaller values.

Surface areas calculated using Eq. 3 from monolayer capacities derived from Eqs. 1 and 4 for all studied materials are shown in Fig. 2.

The agreement between the surface areas calculated using the two approaches above is good and seems to confirm the usefulness of Eq. 4 in estimating surface areas.

The surface areas of the studied biochars measured by nitrogen adsorption markedly increased with the rise in pyrolysis temperature, which has also been frequently reported in the literature^25,26^ and has been mostly attributed to the development of new pores. Below ~ 300 °C, this increase has not been recognised as significant because volatile constituents are blocked in the pores, hindering the formation of new ones^23^. A drastic elevation of the surface area is primarily observed between 400 and 500 °C due to the extensive evacuation of gases, which form deep channels and vascular bundles^15^. Some studies have shown, however, that an increase in surface area does not form a consistent trend over the entire temperature range. Researchers demonstrated that this parameter can only occur up to a certain pyrolysis temperature (500–900 °C, depending on the experiment), above which a decrease is observed due to the onset of ash melting, pore fusion, or destruction^15,23,27^. Moreover, a higher amount of ash in the feedstock has been reported to correlate negatively with the specific surface area in the produced biochar^28^. Modifying biochars with mineral compounds has also resulted in a similar effect of blocked micropores^29^.

In our studies, the nitrogen surface areas of the substrates are lower than those of the biochars. Typically, nitrogen surface areas measured for biochars are extremely low and do not exceed a few square meters per gram, which is consistent with other studies^23^. High surface area is observed only for the acid-washed biochar produced from peat at 600 °C, with intermediate values for biogas plant sludge pyrolyzed at 450 ^o^C and 600 ^o^C. In contrast, surface areas measured by water vapour adsorption are very high and markedly decrease with increasing pyrolysis temperature, which may be partially due to some absorption that can occur in the case of biomass and low-temperature biochars. These materials have a more flexible structure that can stretch and swell in the presence of water vapour molecules, unlike the rigid and porous structures of high-temperature biochars, which primarily act as a matrix for adsorption. It has also been postulated that in the case of biochars, inorganic species constituting ash are considered to create additional adsorption sites for water^30^. The present findings do not support this hypothesis, as the fraction of ash increases with pyrolysis temperature while the water surface area does not.

Changes in the specific surface area of biochars result from chemical processes during the two main stages of pyrolysis. Primary pyrolysis is mostly governed by devolatilisation, which involves the dehydration, decarboxylation and dehydrogenation of the biomass. Secondary pyrolysis encompasses the thermal or catalytic cracking of heavy compounds and char formation. These processes tend to simplify the structure through the degradation of individual organic constituents, with the transformations of hemicellulose, cellulose and lignin being of key importance. Significant changes involving dehydrogenation and decarboxylation reactions appear already at relatively low temperatures of 200–300 °C, typical for torrefaction^31^. Within this range, hemicellulose breaks down to form condensable, light and volatile organics and char^32^. The main structural units of hemicellulose – pentoses and uronic acids – decompose completely at ~ 300 °C. More thermally resistant hexoses require higher temperatures for processing^33^. Cellulose is more stable than hemicellulose due to a higher degree of polymerisation and a partly crystalline structure. Its degradation begins between 300 and 450 °C, involving the breaking of accessible inter- and intra-molecular hydrogen bonds, followed by the more internally placed glycosidic bonds^31,33^. Lignin, a complex, branched macromolecule rich in aromatic components, decomposes across a temperature range of 250–500 °C. The depolymerisation reactions of lignin become particularly intense above 400 °C, producing volatiles abundant in carboxylic acids, phenols, p-cresol, furans, furfural and sugars^34^. As the pyrolysis temperature increases volatilisation decreases, and the share of more thermally stable structures rises. Biochar becomes richer in carbon and poorer in oxygen, while the amount of nitrogen changes only slightly^23^. At temperatures above 500–700 °C, products begin to exhibit significant aromatic character due to the dehydrogenation of hydroaromatics and the condensation of aromatic rings. Analyses reveal the presence of systems with six or more fused benzene rings and graphite structural units^35^.

Minerals in raw materials, concentrated during pyrolysis, can also affect the surface properties of biochars. Depending on the feedstock, metal oxides and salts can comprise 2–25% of the total solid weight. Some cations (particularly K^+^, Na^+^, Ca^2+^ and Mg^2+^) can act as catalysts for critical reactions such as dehydration, demethoxylation, demethylation, decarboxylation and catalytic cracking of volatiles^23,31^. Minerals can also directly affect the morphological properties of biochars. Intermediate liquids formed during the re-condensation of volatilised biomolecules cover the solid particles, promote their agglomeration and facilitate the cross-linking reactions that support char formation. Studies by Boutin et al.^36^ and Carlsson et al.^37^ showed that these films were soluble in water and polar solvents, indicating the formation of a new hydrophilic compound (in contrast to hydrophobic molten cellulose). Fine and loose or molten ash particles can also block the pores of biochar. At higher temperatures, inorganic oxides with low melting points gradually move to the surface of the char, where O-bonds break and the oxides volatilise. This results in the enrichment of free oxygen radicals on the surface and ultimately leads to stable C-O bonds in the char structure^35^.

Changes in surface area can also be connected with structural ordering through graphitisation. This complex process typically occurs at temperatures exceeding 700–800 °C^27^. The graphite structure becomes clearly visible above 1800 °C, while at temperatures above 2100 °C, the surface becomes flat and perfect^38^. In biochar production, the starting temperature for graphitisation can be lowered by the presence of transition metals or inorganic iron compounds^39^. However, oxygen-rich and hydrogen-poor materials are less likely to graphitise due to the formation of cross-linked structures during pyrolysis^26^. The ordering of the structure into graphite-like sheets has been considered competitive with pore structure formation during volatilisation^40^. This process is considered a likely mechanism for thermal annealing, leading to the thermal deactivation of chars. Structural ordering and surface flattening with increasing pyrolysis temperature were confirmed by analyses of surface area as well as by using other research methods. For example, surface smoothing at 500–600 °C and modification by sintering at 1200 °C were observed in SEM micrographs, while elemental analysis and FTIR spectra indicated a reduction of signals associated with the disappearance of functional groups on the biochar surface^41^. Biochar can also lose its high surface area during the ageing process^42^ and with prolonged pyrolysis residence time lasting more than 1–2 h^43^ due to a shift in pore size towards meso- and macro-pores^44^. The presence of air during pyrolysis has also been reported as an important factor contributing to the decrease in BET surface area^44^. Considering all chemical changes of complex organic substances with increasing temperature, one can expect a decrease in their surface area, as revealed by water vapour adsorption rather than nitrogen adsorption measurements.

**Fig. 3:**
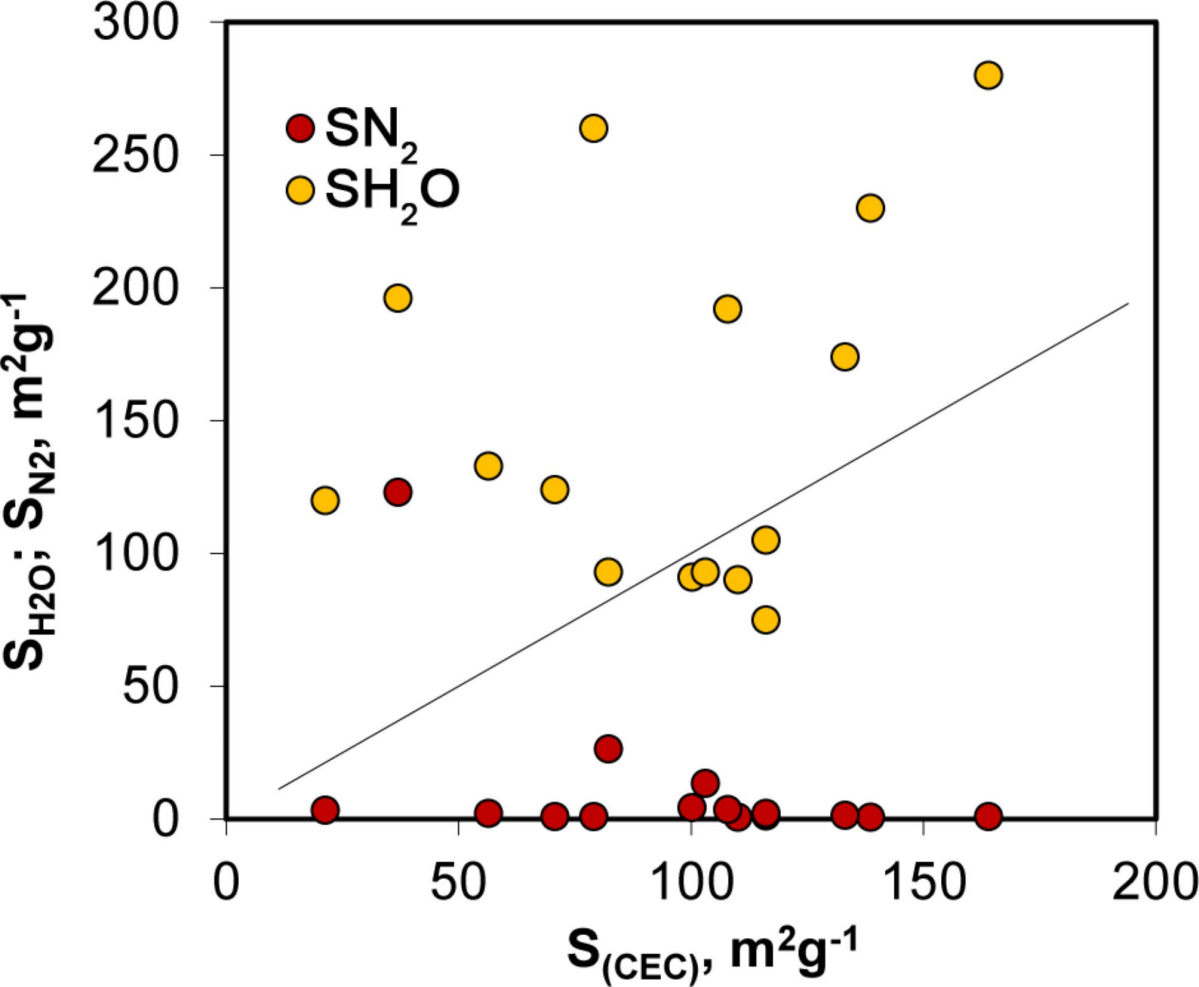
Relationship between surface areas calculated from nitrogen and water vapour adsorption and those derived from CEC values. The solid line represents a 1:1 relationship (S_(CEC)_ = S_(CEC)_), aiding in the visual comparison of all surface area values.

It has been frequently reported that the cation exchange capacity of biochars markedly decreases with heating temperature^45,46^. The CEC is created by acidic surface functional groups, primarily of carboxylic, lactonic and phenolic character^47^. These groups occupy some space on the surface. Thus, by knowing the surface charge, one can roughly estimate the area occupied by acidic surface functional groups by multiplying their amount (surface charge) by the area occupied by a single group. Since variable surface charge data were available for most of the materials studied, the surface occupied by the charged groups was estimated, assuming that the surface charge originates from surface carboxyls that tightly cover the surface. The geometry of the carboxylic group was approximated by that of a formic acid molecule, and the area occupied by a single carboxyl was estimated from the formic acid density. The relationship between surface areas estimated from nitrogen and water adsorption and the CEC values is illustrated in Fig. 3.

One can see that, in most cases, the surface area estimated from water vapour adsorption is sufficient to accommodate all the surface-charged groups responsible for the biochar CEC. In contrast, all but one (P600) nitrogen surface area is far too small to accommodate all the surface functional groups. Therefore, it seems reasonable to rely more on surface areas derived from water vapour adsorption isotherms than on nitrogen ones.

### Adsorption energies

**Fig. 4:**
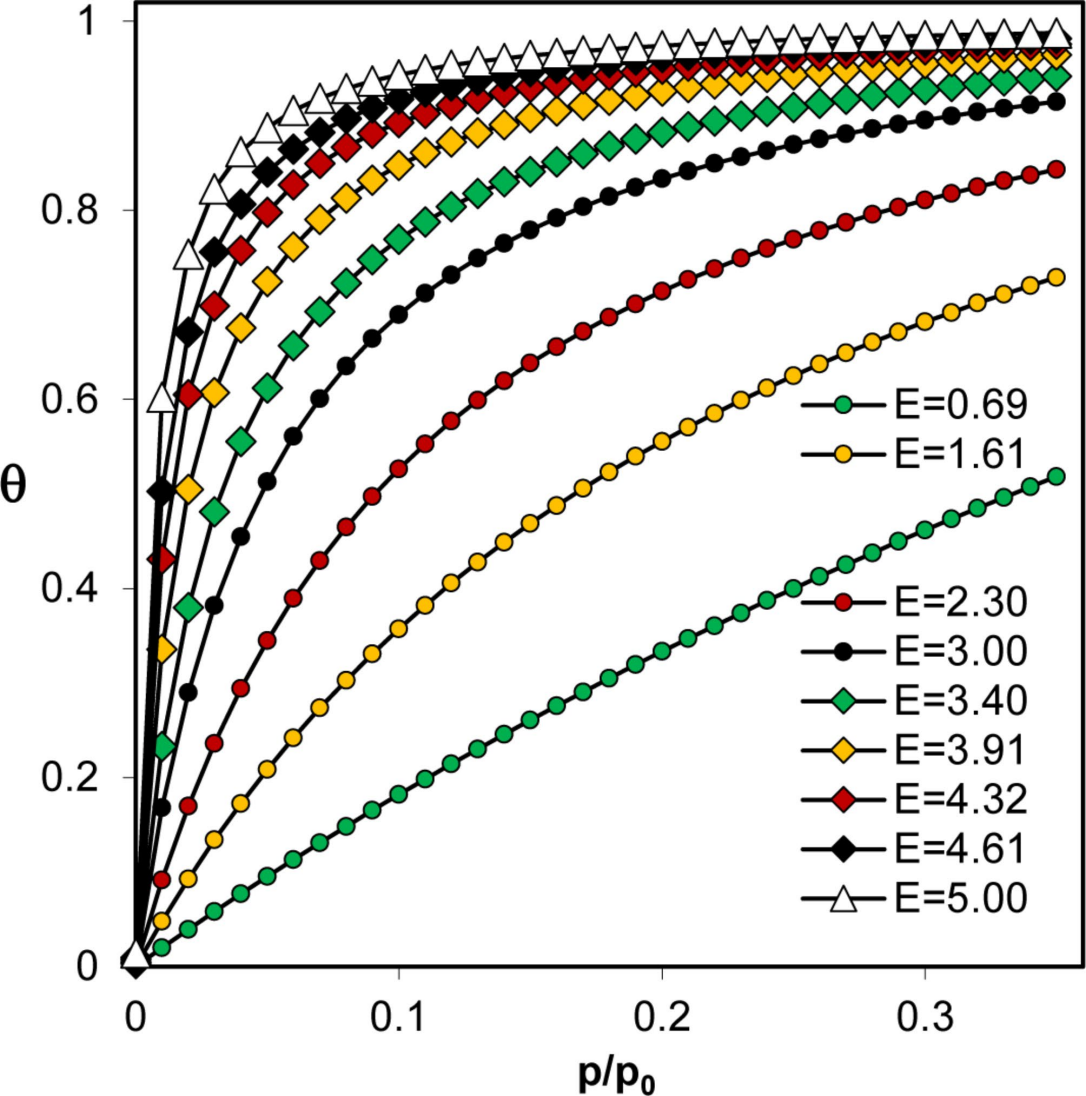
Dependence of BET-simulated monolayer coverage relative adsorbate pressure for various adsorption energies.

Application of the BET model to nitrogen adsorption isotherms of D450, D600, F450, F600, B600, P600 and W600 produced negative intercepts that led to the calculation of negative C values. In such cases, the adsorption energy cannot be evaluated. Interpreting adsorption in energy terms, the very high steepness of these isotherms may suggest very high adsorption energies, as illustrated in Fig. 4, which shows BET-simulated monolayer coverage, Θ = *a*_*mon*_(*p/p*_*0*_)/*a*_*m*_ dependencies on the relative adsorbate pressure for different adsorption energies.

In the case of the BET failure, the adsorption energies should be estimated differently. A simple and reasonable approach has been introduced to evaluate adsorption energies from energy distribution functions, which can be easily obtained from adsorption isotherms^48^. According to the theory of adsorption on heterogeneous surfaces^49,50^, the total monolayer adsorption at a given relative pressure *p/p*_*0*_ can be expressed as a sum of local adsorptions *a*_*i*_ on specific sites of energy *E*_*i*_ = (*E*_*a, i*_ - *E*_*c*_), where *E*_*a, i*_ is the adsorption energy of the *i*-th site:

5 $${a_{mon}}(p/{p_0})\,=\sum\limits_{{i=1}}^{n} {{a_i}(p/{p_0},{E_i})}$$

Thus, the surface coverage, Θ(*p/p*_*0*_), can be written as a sum of the surface coverages of each site, Θ_*i*_(*p/p*_*o*_, *E*_*i*_), weighted by the fractions of sites of distinct energies, *f*(*E*_*i*_):

6 $${\Theta _i}\left( {p/{p_o},{E_i}} \right){\text{ }}=\sum\limits_{{i=1}}^{n} {{\Theta _i}\left( {p/{p_o},{E_i}} \right)/{a_{m,i}} \times \left( {{a_{m,i}}/{a_m}} \right){\text{ }}=} \sum\limits_{{i=1}}^{n} {{\Theta _i}\left( {p/{p_o},{E_i}} \right) \times f\left( {{E_i}} \right),}$$

and the site fractions may be calculated as:

7 $$f\left( {{E_i}} \right){\text{ }}={\text{ }}\left[ {{{\left( {1 - p/{p_{o{\text{ }}i+1}}} \right)}^{1/2}}{\Theta _t}\left( {{E_{i+1}}} \right){\text{ }} - {\text{ }}{{\left( {1 - p/{p_{o{\text{ }}i}}} \right)}^{1/2}}{\Theta _t}\left( {{E_i}} \right)} \right]/\left( {{E_{i+1}} - {E_i}} \right)$$

From *f*(*E*_*i*_) values, the average adsorption energy, *E*_*av*_, can be calculated as:

8 $${E_{av}}=\sum\limits_{{i=1}}^{n} {{E_i}f({E_i})}$$

More details on the above calculations can be found in Jozefaciuk^51^.

**Fig. 5:**
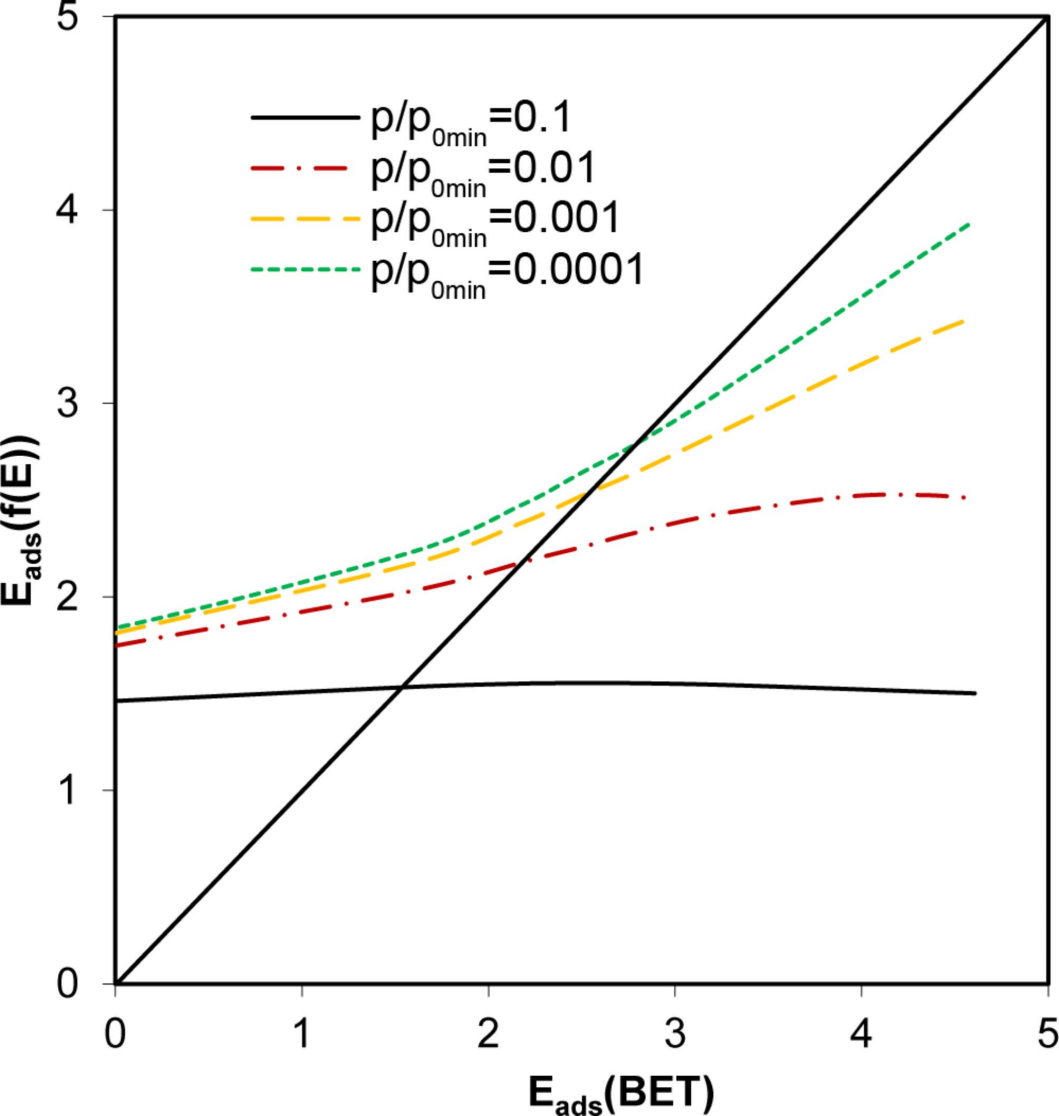
Relationship between adsorption energies calculated from distribution functions at different *p/p*_*0*_ values placed for maximum adsorption energy on the energy calculated from BET equation. The diagonal solid lie represents a 1:1 relationship.

The calculation of adsorption energy distribution functions (*f*(*E*_*i*_) against *E*_*i*_ dependencies) was performed using Eq. 7. Energy values were expressed in units of thermal energy, RT. The lower limit of the scaled energy, (E_a_-E_c_)/RT, was set to 0, which holds for energy adsorption equal to the condensation energy of the vapour. Usually, the maximum energy value should relate to the minimum value of the *p/p*_*0*_ applied. The minimum relative pressure in our water vapour adsorption experiments was around 0.002, which corresponds to an energy adsorption of around − 6. For nitrogen adsorption, it was around 0.001 with an energy of -7. However, these values can be considered only as a first estimate of the maximum energy due to the lack of experimental data at lower relative pressures. We arbitrarily set the maximum energy to -8 for both adsorbates, believing that if there were no sites with higher adsorption energies, the corresponding values of *f*(*E*_*i*_) would be close to or equal to zero. With *f*(*E*_*i*_) values determined, average adsorption energies were calculated from Eq. 8. However, the average energies calculated for the isotherms obeying the BET model never matched those calculated from the respective C(BET) constants. The more or less arbitrary selection of the maximum energy seriously obscured the results. Figure 5 was constructed to illustrate this problem, showing the relationship of adsorption energies calculated from distribution functions at different *p/p*_*0*_ values in relation to the maximum adsorption energy derived from simulated BET adsorption isotherms.

It seems that the usefulness of energy distribution functions for estimating adsorption energies is far from expectations, as there exists only a single value of *p/p*_*0*_ at which adsorption energies calculated from both approaches coincide.

**Fig. 6:**
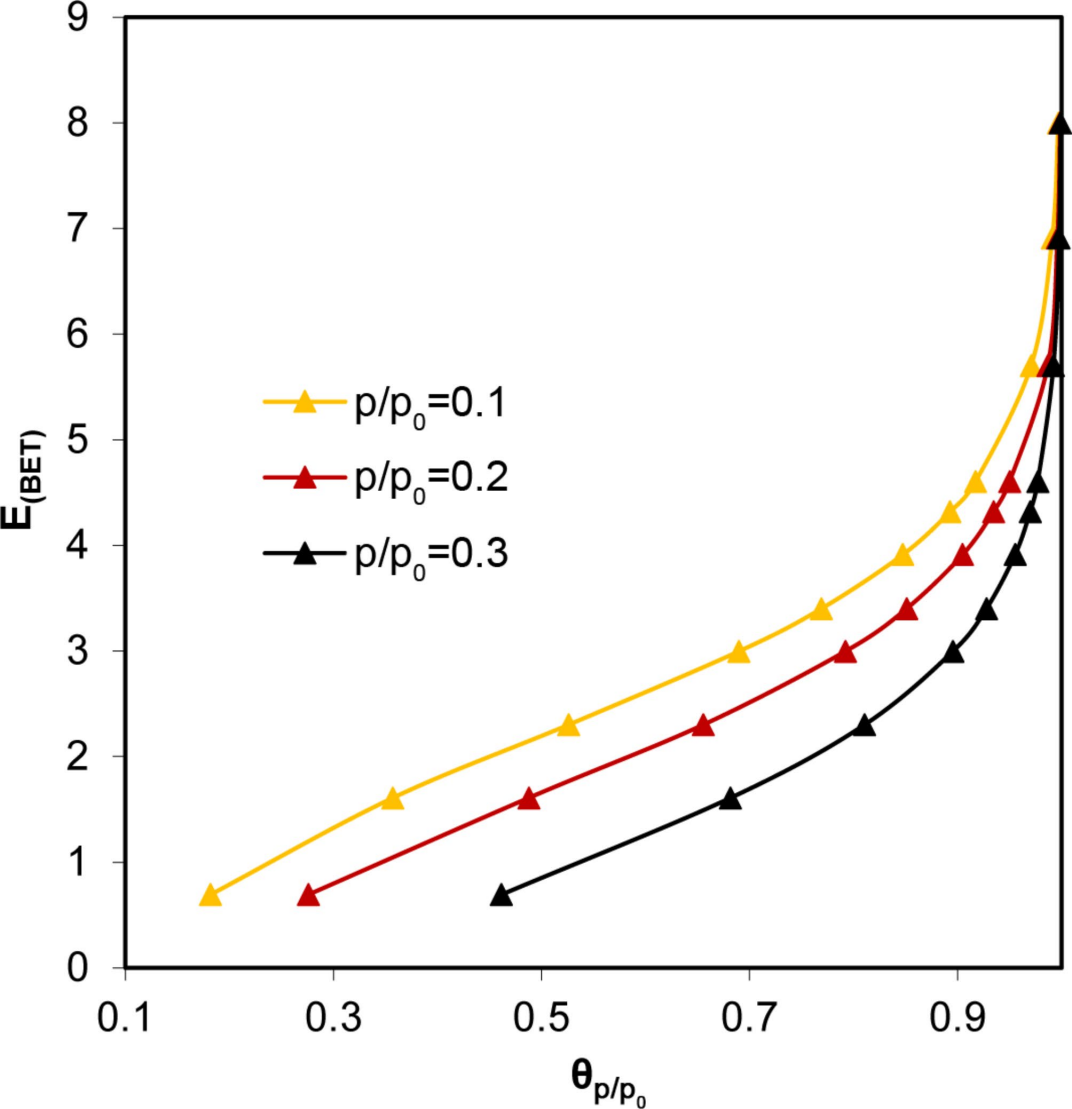
Relationship between adsorption energy calculated from the BET equation on surface coverage at various *p/p*_*0*_ values.

To somehow estimate the adsorption energies from the experimental isotherms with negative C(BET) values, one can rely on the fact that at higher adsorption energies, the surface coverage increases faster with increasing *p/p*_*0*_ values (see Fig. 4). As a measure of the rate of this increase, the ratio of the surface area to monolayer adsorption at any *p/p*_*0*_ can be used (in practice, *p/p*_*0*_ should be located within the range of the BET model applicability, i.e., between 0.05 < *p/p*_*0*_ < 0.35). Figure 6 illustrates the relationship between adsorption energy calculated from the BET equation and surface coverage at three *p/p*_*0*_ values selected from the range in which the BET model is applicable.

**Fig. 7:**
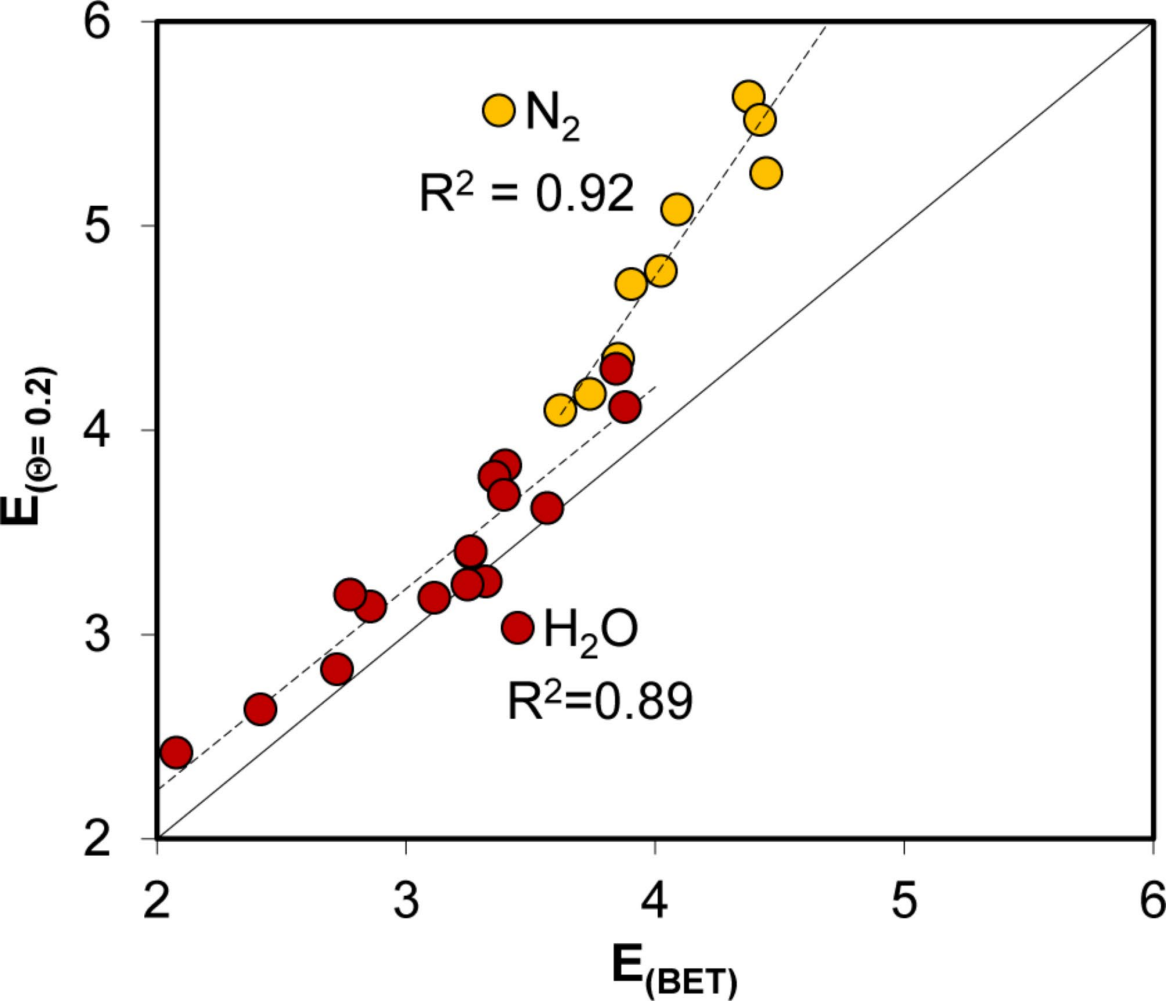
Relationship of adsorption energy calculated from surface coverage at *p/p*_*0*_ = 0.2 on the adsorption energy calculated from BET equation (if applicable). The dashed lines are linear regressions. The solid line is a 1:1 relationship.

Thus, knowing the surface coverage at any *p/p*_*0*,_ one can estimate the adsorption energy from the above plot. Adsorption energies for the studied materials were estimated from surface coverages at *p/p*_*0*_ values of 0.1, 0.2 and 0.3. The monolayer capacity values of *a*_*mon, max*_ (Eq. 4) were used. By knowing the Θ values and applying linear interpolation to the curves from Fig. 6, the adsorption energies were estimated. The energy values estimated from surface coverages at different *p/p*_*0*_ values were similar; however, the best results were achieved for *p/p*_*0*_ = 0.2 (for nitrogen, the adsorption energies derived from Θ at *p/p*_*0*_ = 0.3 deviated the most from the others, and for water vapour adsorption, those derived from Θ at *p/p*_*0*_ = 0.1). The relationship between the adsorption energy calculated from surface coverage at *p/p*_*0*_ = 0.2 and the adsorption energy calculated from the BET equation for the studied materials is shown in Fig. 7.

For water vapour adsorption, the energies derived from surface coverage are very close to the BET values, whereas, for nitrogen adsorption, these appear to be significantly higher.

The studied materials’ adsorption energies estimated from nitrogen and water vapour adsorption isotherms are listed in Table 3. Generally, the adsorption energies were estimated using the BET model. In cases where it fails, the data estimated from surface coverage at *p/p*_*0*_ = 0.2 are presented.

**Table 3:** Adsorption energies of the studied materials.

	F	F300	F450	F600	D	D300	D450	D600	B	B300	B450		B600	*P*	P300	P600	W	W300	W600
*N_2_	4.4	4.4	***4.6***	***4.9***	4.1	4.02	***4.6***	***5.2***	3.6	3.7	4.4		***4.5***	3.9	3.8	***5.1***	4.6	4.8	4.7
H_2_O	2.7	2.1	2.4	2.9	3.6	3.3	3.3	2.8	5.8	3.1	3.3		3.4	3.9	3.8	3.4	3.3	3.4	2.7

The standard deviation did not exceed 3%. *Data in bold italic are derived from monolayer coverage at *p/p*_*0*_ = 0.2.

Adsorption energies estimated from nitrogen adsorption are generally, markedly higher than those calculated from water vapour adsorption. This is somewhat surprising since the forces of interaction between nitrogen molecules, which exhibit only a low quadrupole moment, and the surface are markedly weaker than those of water molecules, which possess a high dipole moment. An increase in nitrogen adsorption energy with pyrolysis temperature is also surprising due to the consecutive removal of polar functional groups, which constitute adsorption centres of high energy, from the surface during the heating process. Water vapour adsorption energies exhibit the opposite, though slightly less evident, trends with pyrolysis temperature. They appear to be highest for raw substrates with highly polar surfaces and lower for all biochars, reflecting the chemical changes during heating.

### General remarks

Our manuscript is addressed to scientists, researchers and industrial centres dealing with the management and modification of biomass properties, as well as the products of its thermal treatment, which were not burned to ash but were heated in conditions of oxygen deficiency sufficiently to become biochar.

In the present studies, the biomass feedstock composition and pyrolysis temperature were the major factors influencing biochar surface area, as frequently reported by other authors^15,52^. These factors also affected the adsorption energy.

Water vapour adsorption on biochars appears to be primarily driven by physical interactions. In all studied cases, it obeyed the BET model, which was not correct for several nitrogen adsorption isotherms. In many cases, the nitrogen adsorption isotherms were very steep at the beginning of adsorption, which has been frequently interpreted in terms of fine nanopore filling. Therefore, an increase in nitrogen adsorption (e.g., due to an increase in biochar pyrolysis temperature) has been commonly interpreted as an increase in fine pores and surface area. An alternative interpretation of nitrogen adsorption isotherms in terms of adsorption energy gives extremely high nitrogen adsorption energies, much higher than those for water vapour adsorption. This seems somewhat strange because the interaction forces between water molecules and the biochar surface should be stronger, as these involve strong dipole interactions.

Comparing the adsorption quantities of nitrogen and water, one can conclude that highly energetic sites and/or fine nanopores detected by nitrogen are largely undetected by water vapour adsorption, as the latter is usually hundreds of times higher.

It is particularly important to address all environmental and sorption applications of biochars in relation to their surface areas measured by water vapour adsorption. Water sorption is crucial for understanding soil water retention, particularly in conditions of low moisture. Additionally, the surface area estimated from water vapour adsorption appears to be able to capable of carrying surface charges responsible for the cation exchange capacity of biochars. The CEC decreases with pyrolysis temperature, paralleling the water surface area, due to the reduction of the content of oxygenated functional groups on the biochars’ surface.

## Conclusions

Water adsorption appears to be a much better tool for estimating surface areas and adsorption energies of biochars than nitrogen adsorption. This main conclusion has been drawn because:

In many cases, nitrogen adsorption isotherms yielded negative values for the intercepts of the linear BET equation, which is theoretically restricted. This leads to the impossibility of estimating adsorption energy. Therefore an alternative method for estimating adsorption energy based on surface coverage was proposed.

The maximum amount of the adsorbate covering the surface as a monolayer frequently exceeded the monolayer capacity, which was mainly observed for nitrogen adsorption isotherms. In such cases, an alternative model for estimating biochar surface area was proposed.

The observed decreases in surface areas and adsorption energies from water vapor adsorption align with the expected chemical changes in organic surfaces during heating. Conversely, the opposite changes were observed for nitrogen adsorption.

The surface areas were frequently too small to support surface charged groups responsible for biochar cation exchange capacity. This was particularly pronounced for nitrogen surface areas.

In most cases, nitrogen adsorption was about two orders of magnitude smaller than water vapour adsorption. This suggests that nitrogen molecules are unable to detect a large part of the surface area that is accessible only through pores with very fine entrances.

## Data Availability

Data sets generated during the current study are available from the corresponding author upon reasonable request.
